# Icotinib Is an Active Treatment of Non-Small-Cell Lung Cancer: A Retrospective Study

**DOI:** 10.1371/journal.pone.0095897

**Published:** 2014-05-16

**Authors:** Xiaofeng Chen, Quan Zhu, Yiqian Liu, Ping Liu, Yongmei Yin, Renhua Guo, Kaihua Lu, Yanhong Gu, Lianke Liu, Jinghua Wang, Zhaoxia Wang, Oluf Dimitri Røe, Yongqian Shu, Lingjun Zhu

**Affiliations:** 1 Department of Oncology, the First Affiliated Hospital of Nanjing Medical University, Nanjing, China; 2 Department of Cardiothoracic Surgery, the First Affiliated Hospital of Nanjing Medical University, Nanjing, China; 3 Department of Oncology, Jinling Hospital, Nanjing University School of Medicine, Nanjing, China; 4 Department of Oncology, the Second Affiliated Hospital of Nanjing Medical University, Nanjing, China; 5 Department of Cancer Research and Molecular Medicine, Norwegian University of Science and Technology, Trondheim, Norway; 6 Norwegian University of Science and Technology, Trondheim, Norway, Cancer Clinic, Levanger Hospital, Nord-Trøndelag Health Trust, Levanger, Norway; H. Lee Moffitt Cancer Center & Research Institute, United States of America

## Abstract

**Background:**

Icotinib hydrochloride is a novel epidermal growth factor receptor (EGFR) tyrosine kinase inhibitor (TKI) with preclinical and clinical activity in non-small cell lung cancer (NSCLC). This retrospective analysis was performed to assess the efficacy of icotinib on patients with non-small-cell lung cancer (NSCLC).

**Methods:**

82 consecutive patients treated with icotinib as first (n = 24) or second/third line (n = 58) treatment at three hospitals in Nanjing were enrolled into our retrospective research. The Response Evaluation Criteria in Solid Tumors (RECIST) was used to evaluate the tumor responses and the progression-free survival (PFS) and overall survival (OS) was evaluated by the Kaplan-Meier method.

**Results:**

Median PFS was 4.0 months (95% CI 2.311–5.689). Median OS was 11.0 months (95% CI 8.537–13.463) in this cohort. Median PFS for first and second/third line were 7.0 months (95% CI 2.151–11.8) and 3.0 months (95% CI 1.042–4.958), respectively. Median OS for first and second/third line were 13.0 months (95% CI 10.305–15.695) and 10.0 months (95% CI 7.295–12.70), respectively. In patients with EGFR mutation (n = 19), icotinib significantly reduced the risk of progression (HR 0.36, 95% CI 0.18–0.70, *p* = 0.003) and death (HR 0.10, 95% CI 0.02–0.42, *p* = 0.002) compared with those EGFR status unknown (n = 63). The most common adverse events were acne-like rash (39.0%) and diarrhea (20.7%).

**Conclusions:**

Icotinib is active in the treatment of patients with NSCLC both in first or second/third line, especially in those patients harbouring EGFR mutations, with an acceptable adverse event profile.

## Introduction

The epidermal-growth-factor receptor (EGFR) is part of a signalling pathway that regulates tumor cell proliferation, invasion, angiogenesis, metastasis, and apoptosis [1]. EGFR tyrosine-kinase inhibitors (TKIs) can inhibit tumor cells by blocking the EGFR signaling via binding to ATP binding site on the tyrosine kinase domain of the EGFR. Activating EGFR mutations are found in about 60% of lung adenocarcinomas in the East Asian population and nonsmoker or former light smoker [2]. Numerous phase III studies have shown that EGFR TKIs such as gefitinib or erlotinib have strong anti-tumor activity and increase survival in patients with NSCLC harboring activating EGFR mutation not only in second and third line [3], [4], [5], but also in first line [2], [6], [7] and maintenance treatment [8], [9], [10].

Icotinib hydrochloride is a new type of small molecule EGFR-TKI, developed and patented by Zhejiang BetaPharma Co., Ltd. (Hangzhou, Zhejiang, China, Patent No. WO2003082830). Its chemical name is 4-((3-ethynylphenyl) amino)-6, 7-benzo-12-crown-4- quinazoline hydrochloride. The molecular formula is C22H21N3O4·HCl, with a small molecular weight of 427.88. Preclinical studies showed that icotinib is a potent and specific EGFR TKI. *In vitro,* icotinib could significantly inhibit the EGFR tyrosine kinase activity at a concentration of 0.5 µM including the EGFR (91%), EGFR (L858R) (99%), EGFR (L861Q) (96%), EGFR (T790M) (61%) and EGFR (T790M, L858R) (61%) [11]. In EGFR-mutated lung cancer cell lines (PC-9 and HCC827), icotinib showed similar anti-tumor effect compared with gefitinib [12]. *In vivo*, icotinib strongly inhibited the tumor growth in several xenograft models in a dose related manner [11].

Two phase I studies evaluated the safety and tolerability of icotinib in Chinese patients with NSCLC and other solid tumors [13], [14]. Generally, icotinib showed favorable safety and tolerability. Mild and reversible rash, diarrhea and nausea were the main adverse events. Notably, positive anti-tumor activities were observed in patients with advanced NSCLC. A dose of 125 mg or 150 mg q8h/day was recommended for phase II/III studies.

A randomized, double-blind phase III study [15] was carried out to compare the efficacy and safety of icotinib with gefitinib in NSCLC patients previously treated with chemotherapy (ICOGEN) [16]. A total of 399 patients with advanced NSCLC who had progressed after one or two lines of chemotherapies were randomized to receive icotinib (n = 200, 125 mg q8h) or gefitinib (n = 199, 250 mg qd). Compared with gefitinib, icotinib provided similar median progression-free survival (PFS, icotinib vs. gefitinib: 4.6 months vs. 3.4 months, HR 0.84, 95% CI 0.67–1.05) and median overall survival benefit (OS, icotinib vs. gefitinib: 13.3 months vs. 13.9 months, HR 0.90, 95% CI 0.79–1.02). As with gefitinib, biomarker analysis revealed that EGFR mutation status was the strongest predictor for icotinib. The PFS in patients treated with icotinib was 7.8 months in EGFR mutant subgroup and 2.3 months in EGFR wild type population.

Based on the encouraging results of ICOGEN reported in ASCO annual meeting in 2011, icotinib was approved for the second or third line treatment of advanced NSCLC by the State Food and Drug Administration of China. Gefitinib and erlotinib have shown good efficacy as first line treatment in clinically selected patients, while icotinib has the similar molecular structure with gefitinib and erlotinib. In addition, icotinib is much cheaper than gefitinib or erlotinib in China. Thus icotinib was also an alternative choice for first line treatment in the clinic. Here, we retrospectively analyzed the efficacy of icotinib on the treatment of advanced NSCLC patients as first line or second/third line treatment in clinical practice.

## Patients and Methods

### Ethics Statement

This study was approved by the three hospitals Review Boards: the First and Second Affiliated Hospital of Nanjing Medical University, and Jinling Hospital of Nanjing University School of Medicine. Patient records were anonymized and de-identified prior to analysis.

### Patients

We conducted a retrospective search of the medical records in the First and Second Affiliated Hospital of Nanjing Medical University, and Jinling Hospital of Nanjing University School of Medicine, from July 2011 to February 2013. Excluding the patients received icotinib as forth or later lines treatment and those whose clinical data were not available, 82 patients who received icotinib as first line (n = 24) or second/third line (n = 58) treatment were enrolled into our retrospective search. All these patients received oral icotinib at a dose of 125 mg q8h/day continuously until either a disease progression (radiographic or obvious clinical) or severe toxicity was observed. No other chemotherapeutic drug was administered concurrently with icotinib treatment.

### Assessment of the Efficacy and Adverse Events

The Response Evaluation Criteria in Solid Tumors (RECIST) was used to evaluate the tumor responses [17]. The evaluation was from the patients’ original medical records and in some uncertain cases, was re-evalatued by two radiologist (H.L & Y.J, radiology department in the First Affiliated Hospital of Nanjing Medical University). Objective response comprises complete response (CR) and partial response (PR). Disease control was defined as CR, PR or stable disease (SD). Generally, after starting with icotinib, the first evaluation was performed at one month, and then assessed every two months or at overt signs of progression. PFS was defined as the period from the initial administration of icotinib to tumor progression or death of any cause (calculated according to the event occurred firstly). OS was defined as the span between the start of icotinib and the date of death. Adverse events were assessed according to Common Terminology Criteria for Adverse Events of the National Cancer Institute (version 3.0) [18]. Clinical data and outcomes were collected by patient medical records search, consulting the doctors in charge, interview in the clinic and phone calls to the patients or their relatives.

### Statistical Analysis

The chi-square (χ2) test was used for intergroup comparisons of response rate and disease control rate at a significance level of 5% (α = 0.05, two-sided). PFS and OS were obtained using the Kaplan-Meier method. Log-rank test was applied to compare the significance between groups. Multivariate Cox-regression model was also used to detect the hazard ratios. SPSS software package (SPSS 17.0 Inc., Chicago, IL, USA) was applied.

## Results

### Patients Characteristics

A total of 82 patients who received icotinib as first (n = 24) or second/third (n = 58) line treatment were enrolled in this retrospective analysis. The median age of the population was 64 years. All patients were Chinese. Most patients were non-smokers, had adenocarcinoma, stage IV disease and relatively low performance status (Table 1). EGFR sensitive mutation was found in 19 patients (8 in first line and 11 in second/third line) while unknown in the remaining cases. This may due to the lack of proper tissue and some patients were reluctant to receive the gene detection. Five patients had diagnosed brain metastasis before they received icotinib. In the second/third lines subset, the most frequently used chemotherapy regimens for first/second line treatment were pemetrexed+cisplatin/carboplatin, paclitaxel+cisplatin/carboplatin, docetaxel+cisplatin/carboplatin and gemcitabine+cisplatin. Two patients received gefitinib as first line treatment.

**Table 1:** Patient characteristics.

	First line N = 24	Second/third line N = 58	Total N = 82 (%)
Median age	64(37–79)	65(30–80)	64
Sex			
Male	9	32	41 (50)
Female	15	26	41 (50)
ECOG			
0–1	7	20	27 (32.9)
2	14	28	42 (51.2)
3	3	10	13 (15.8)
Smoking history			
Non-smoker	14	36	50 (60.9)
Smoker	10	22	32 (39.1)
EGFR mutation			
Del 19	5	7	12 (14.6)
L858R	3	4	7 (8.5)
Not available	16	47	62 (36.9)
Clinical stage			
IIIb	2	7	9 (11)
IV	22	51	73 (89)
Pathological type			
Adeno	23	52	75 (91)
Squamous	1	5	6 (7.3)
Other	0	1	1 (1.7)

### Response Rate

The CR, PR and SD of the whole group were 1%, 22% and 41%, respectively, a 65% overall disease control rate. Twelve of 19 patients (63%) bearing EGFR mutation experienced an objective response, which was significantly higher than that in patients with EGFR status unknown (7 out of 63, 11%). Higher response rates were observed in EGFR mutation positive patients in first as well as second/third line, while a higher disease control rate was only seen in the first line subset (Table 2).

**Table 2:** Best response according to RECIST 1.0.

	First line n = 24			Second/third line n = 58			All patients N = 82			Total response
	EGFRmut+N = 8	EGFR statusunknown N = 16	P*	EGFRmut+N = 11	EGFR statusunknown N = 47	P**	EGFRmut+N = 19	EGFR statusunknown N = 63	P***	
CR	0 (0%)	0 (0%)	/	1 (9%)	0 (0%)	0.190	1 (5%)	0 (0%)	0.232	1 (1%)
PR	6 (75%)	2 (13%)	0.005	5 (45%)	5 (11%)	0.013	11(58%)	7 (11%)	0.000	18 (22%)
SD	2 (25%)	7 (44%)	0.380	3 (27%)	22 (47%)	0.320	5 (26%)	29 (46%)	0.184	34 (41%)
DC	8 (100%)	9 (57%)	0.034	9 (82%)	27 (53%)	0.178	17 (89%)	36 (57%)	0.013	53 (65%)
PD	0 (0%)	7 (44%)	0.034	2 (18%)	20 (43%)	0.178	2 (11%)	27 (43%)	0.013	29 (35%)

Notes: CR, complete response; PR, partial response; SD, stable disease; DC, disease control; PD, progressive disease.

*EGFR mut vs EGFR unknown in first line icotinib treatment,

**EGFR mut vs EGFR unknown in second/third line icotinib treatment,

***EGFR mut vs EGFR unknown in all patients who received icotinib treatment.

### Progression-free Survival and Overall Survival

**Figure 1:**
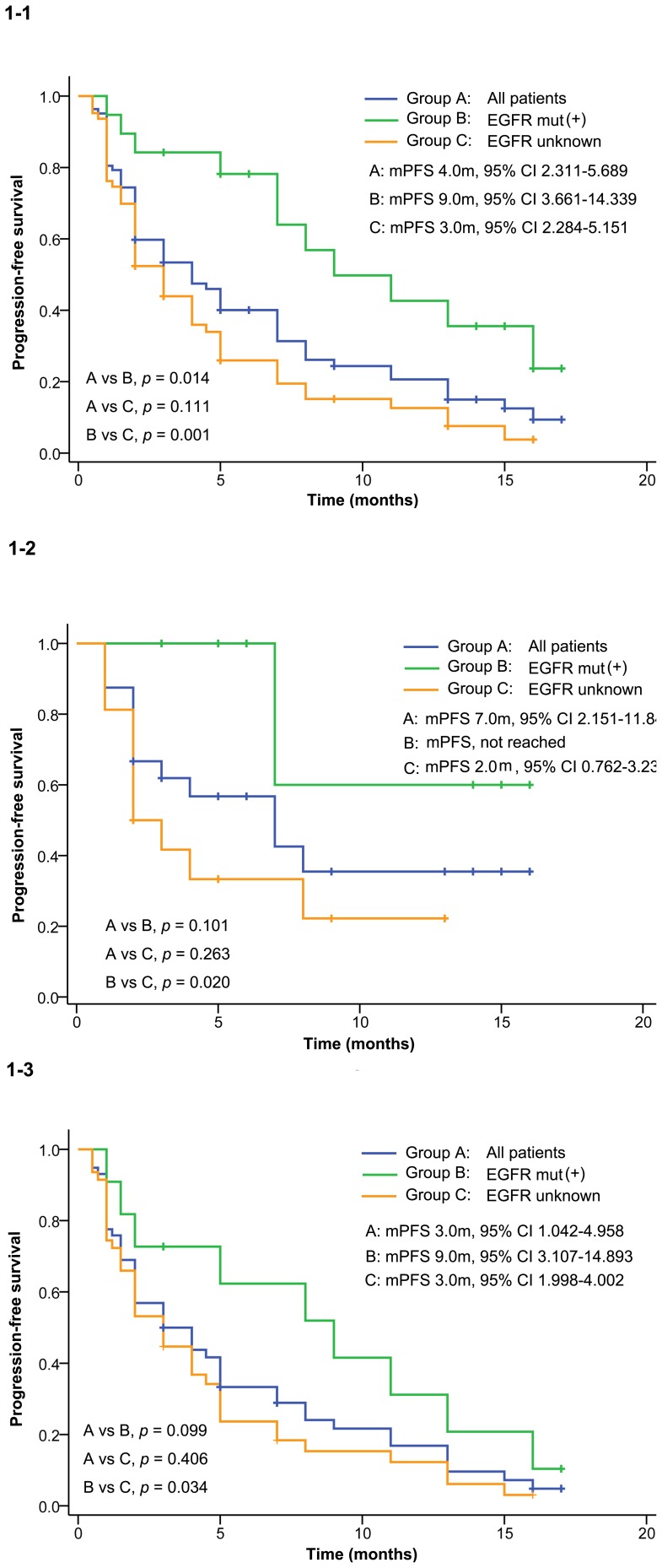
Progression-free survival. 1–1 shows the PFS in all patients in this study, 1–2 shows the PFS in first line subset, 1–3 shows the PFS in second/third line subset.

**Figure 2:**
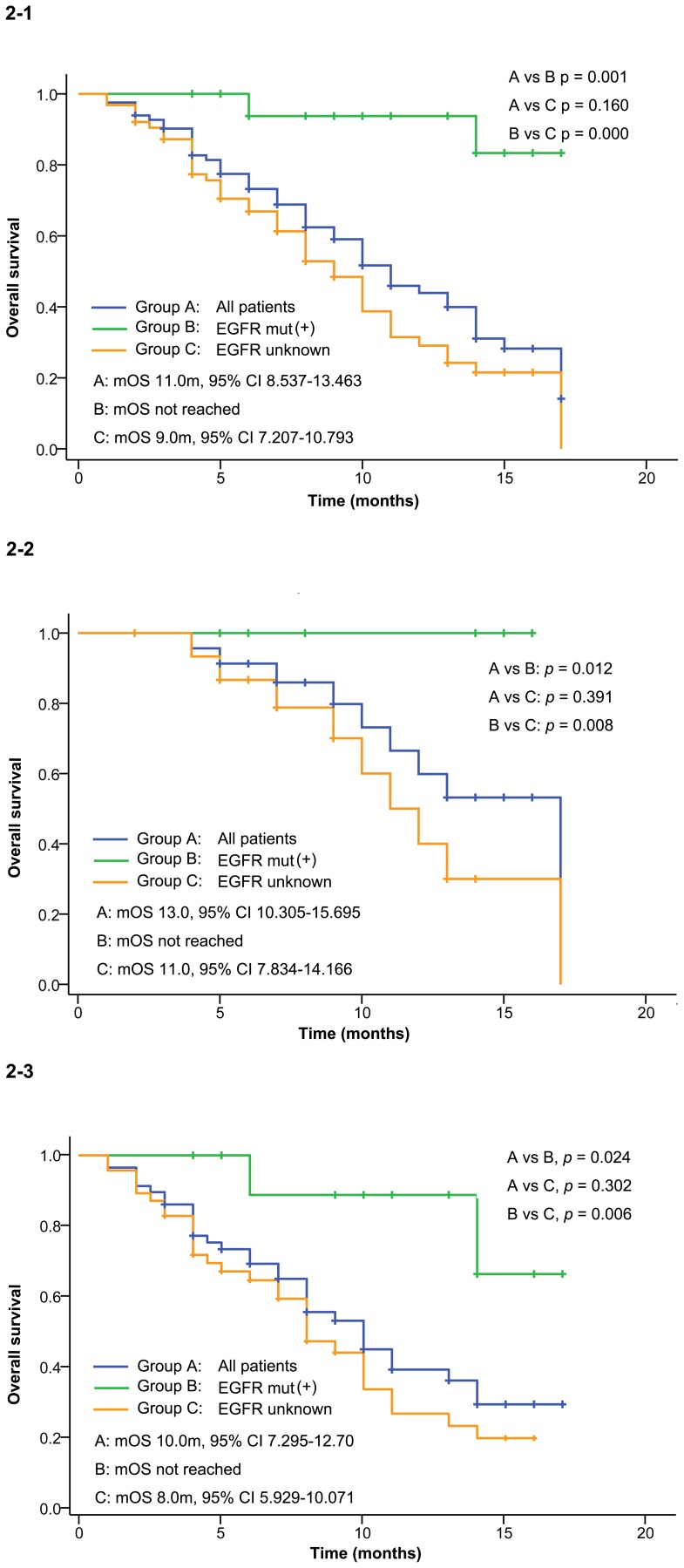
Overall survival. 2–1 shows the OS in all patients in this study, 2–2 shows the OS in first line subset, 2–3 shows the OS in second/third line subset.

Median progression-free survival for the 82 patients was 4.0 months (95% CI 2.311–5.689). Patients bearing EGFR mutation had a significantly longer PFS (9.0 months, 95% CI 3.661–14.339) than those with EGFR status unknown (3.0 month, 95% CI 2.284–5.151, *p* = 0.001) or the whole population (*p* = 0.014) (Figure 1–1). Median progression-free survival was 7.0 months (95% CI 2.151–11.849; Figure 1–2) in first line subset and 3.0 months (95% CI 1.042–4.958, Figure 1–3) in second/third lines subset. Consistently, patients with EGFR sensitive mutation had longer PFS both in first line (*p* = 0.020) and in second/third line subgroups (*p* = 0.034) compared with those whose EGFR status were unknown. Multivariate analysis using Cox regression model indicated that icotinib significantly decreased the risk of progression in patients with EGFR mutation compared with EGFR status unkown (HR, 0.36, 95% CI 0.18–0.70, *p* = 0.003). In patients with EGFR mutation icotinib showed decreased risk of progression in the first line subset, (HR, 0.21, 95% CI,0.05–0.97, *p* = 0.046), while insignificant/trend of risk reduction in the second/third line subset (HR, 0.51, 95% CI 0.24–1.07, *p* = 0.073).

Median overall survival for all patients was 11.0 months (95% CI 8.537–13.463, Figure 2–1). Consistent with the results in PFS, patients with EGFR mutation had statistically longer OS (not reached) than those with EGFR status unknown (mOS 9.0 months, 95% CI 7.207–10.793, *p* = 0.000) or the whole population (*p* = 0.001). This superiority was consistently observed both in first and second/third line subsets. The median overall survival was 13.0 months (95% CI 10.305–15.695,) in the first line treatment subgroup (Figure 2–2), and 10.0 months (95% CI 7.295–12.70) in the second/third line (Figure 2–3). Multivariate analysis showed that icotinib significantly reduced the risk of death in EGFR mutation patients (HR 0.10, 95% CI 0.02–0.42, *p* = 0.002). This significant reduction were found both in first line (HR 0.27, 95% CI 0.10–0.87, *p* = 0.032) and second/third line subsets (HR 0.18, 95% CI 0.04–0.75, *p* = 0.018).

### Adverse Events

Drug related adverse event were registered in 61 of 82 patients (74.3%, Table 3). The most common adverse events were skin-related events and diarrhea. The incidence of acne-like rash and diarrhea were 39.0% and 20.7% respectively. Other common adverse events include dry skin, oral ulcer, nausea, fatigue, elevated ALT/AST, and leukopenia. However, most of the adverse events were grade 1 or 2, and only two grade 3 acne-like rashes were recorded. No possible drug-related interstitial lung disease and drug related death was noted and no patient had dose reduction due to the adverse events. The two patients with grade 3 rash refused to reduce the dose of icotinib, and the symptoms were alleviated through symptomatic treatment.

**Table 3:** Summary of the treatment-related adverse events.

Adverse events	Grade 1	Grade 2	Grade 3	Grade 4	Total (%)
Acne-like rash	24 (29.3%)	6 (7.3%)	2 (2.4%)	0	32 (39.0%)
Dry skin	7 (8.5%)	2 (2.4%)	0	0	9 (11.0%)
Oral ulcer	4 (4.9%)	1 (1.2%)	0	0	5 (6.1%)
Diarrhea	12 (14.6%)	5 (6.1%)	0	0	17 (20.7%)
Nausea	5 (6.1%)	0	0	0	5 (6.1%)
Fatigue	3 (3.7%)	2 (2.4%)	0	0	5 (6.1%)
Elevated ALT/AST	6 (7.3%)	2 (2.4%)	0	0	8 (9.8%)
Leucopenia/neutropenia	2 (2.4%)	0	0	0	2 (2.4%)

## Discussion

Since the appearance of gefitinib and erlotinib, a novel TKI inhibitor, icotinib, has recently showed effect in NSCLC [16]. This relatively small retrospective study of a novel EGFR inhibitor, icotinib, in unselected NSCLC patients from three hospitals showed an encouraging disease control rate (65%), progression-free survival (4.0 m) and overall survival (11.0 m). We observed that a large proportion of patients who responded to icotinib and longer PFS were observed in patients with EGFR mutation. Although the OS in patients with EGFR mutation was not reached, it was statistically longer than that in patients with EGFR status unknown. These results suggest activity of icotinib in non-small cell lung cancer, and robust activity of icotinib in patients with EGFR mutation.

In the second/third line subset (n = 58), the PFS were 3.0 m for all patients, 9.0 m for EGFR mutation patients and 3.0 m for EGFR status unknown patients. The OS was 10.0 for all patients, while the OS for EGFR mutation patients was not reached. These results were similar with those reported in ICOGEN. In the ICOGEN study, patients with NSCLC that progressed after one or two lines of chemotherapies were randomized to receive icotinib (150 mg tid) or gefitinib (250 mg qd). In the icotinib group, the median PFS were 4.6 m for all patients and 7.8 m in patients with EGFR mutation, and the OS was 13.3 months, comparable with the results of gefitinib group [16], [19]. Gefitinib and erlotinib has been widely tested as second/third line treatment for lung cancer in a series of prospective studies, such as INTREST [3], ISEL [5], TITAN [4] and BR.21 study [20]. The PFS of tyrosine kinase inhibitors (TKIs) groups in these studies ranged from 6.3 weeks to 3.3 months, and the OS ranged from 5.3 to 7.6 months. Our retrospective study, based on the real clinical practice, together with results from ICOGEN, showed that icotinib produced a comparable benefit with gefitinib or erlotinib, and indicated that it could be an alternative choice for patients in second/third line treatment.

In first line subset, the PFS and OS were 7.0 m and 13.0 m for all patients, 2.0 m and 11.0 m for EGFR status unknown patients respectively. PFS and OS for EGFR mutation patients were not mature. Currently, no other study reports the efficacy of icotinib as first line treatment. Three ongoing prospective trials will give more information about the activity of icotinib in first line (NCT01646450, NCT01665417, NCT01719536). Patients in our study were all Asian, 96% of them had adenocarcinoma, 63% were female, and 58% were non-smoker. These clinical features indicate that there may be a relative high rate of EGFR mutation. The PFS in this study was very similar with that in patients treated with gefitinib in IPASS study [2], which enrolled patients with clinical enrichment of EGFR mutation, including East Asian, female, non-smoker or light smoker and adenocarcinoma. Compared with those data in gefitinib and erlotinib studies, the PFS and OS in this study were numerically good, considering that there were 55 patients (67.1%) had a PS ≥2 in this population. However, one should note that this is a retrospective study and the assessment bias caused by clinical doctors may exist while the abovementioned studies on gefitinib and erlotinib were all prospective randomized controlled trials. In addition, the number of cases in this study was small, thus the results may be heavily influenced by individual cases, especially those with EGFR active mutation.

EGFR mutation is a prognostic factor regardless of the treatment with EGFR TKIs [21] or chemotherapy [22]. Moreover, it is also the strongest predictive factor for the efficacy of EGFR TKIs [2], Similarly, in the current study, significantly better response rates and survival results were noted in patients with EGFR mutation, compared with those with EGFR status unknown, in both first line and second/third lines subsets. Ren Guanjun et al [23] analysed the relationship between EGFR mutations and the efficacy of icotinib in patients enrolled in a phase I study, the results showed that EGFR exon 19 deletions and exon 21 point mutation are predictive biomarkers for response to icotinib hydrochloride as second line or subsequent lines of treatment. In the ICOGEN study, PFS and OS were longer in patients with EGFR mutation (7.8 m and 20.9 m, respectively) than those with EGFR mutation-negative (2.3 m and 7.8 m, respectively). Thus, EGFR mutation status is the strongest predictor in identifying which patients are most likely to benefit from icotinib. Based on these results and previous evidence from gefitinib and erlotinib, EGFR mutation should be detected when considering the use of icotinib.

The adverse events in this study was similar with those observed in ICOGEN study, while numerically less than those with gefitinib [2], [24], [25] and erlotinib [6], [26]. Although skin related events and diarrhea were common, most of the events were grade 1, and no patient needed dose reduction. However, the available data for both the efficacy and safety of icotinib are limited, and more studies are needed to address the role of icotinib in the treatment of non-small cell lung cancer. Besides the ICOGEN study, 13 prospective trials about icotinib are now active.

The key weakness of this report is its retrospective nature. The evaluation of efficacy and toxicity was not predefined. Another weakness is that the sample was small, especially for the first line subset. In addition, only a small portion of patients had EGFR status known both in first and second/third line.

In summary, a novel TKI, icotinib showed clinically meaningful activity in the treatment of patients with lung adenocarcinoma, especially in those patients harbouring EGFR mutations, with an acceptable adverse event profile. The outcomes of the ongoing trials on icotinib will give more evidence for the value of icotinib in the treatment of NSCLC.
